# Regeneration of the Femoral Passage after Extended Resections of the Hip

**Published:** 1919-01

**Authors:** 


**On the Regeneration of the Femoral Passage after Extended Resections of the Hip.** By M. Broca. *Bulletin de la Société de Chirurgie de Paris*, May 29, 1918.

Speaking of M. Leriche's process for applying to the hip the principles of sub-capsulo periosteal resections, and for obtaining widely spread regenerations, Broca insisted upon the necessity of " exaggerating the Ollier practice ", that is, cutting forcibly with a sharp rugine into the bone substance, thus obtaining osteogenesis.

Broca stated the conflicting theories regarding bone regeneration. Larghi's supposition is that the periosteum, separated and isolated from the subjacent bones, is capable of reproducing bone subtance. The doctrine of abscission upholds that " every portion of a healthy bone covered with an adherent periosteum helps in the regeneration of the bones by the proliferation of its subperiosteal cells ", and that this work of osteogenesis, when the bone has been broken, injured, hollowed or cauterized, is of remarkable activity.

Broca then proceeded to discuss various theories of osteogenesis, notably as to whether the periosteum itself contained the regenerating elements. He concluded that there is a cellular substance between the periosteum and the bone capable of ossification, but, as Robin says, " the periosteum is a fibrous laminous membrane, which in itself cannot produce bone ".

In practicing exaggerated esquillectomy the subperiosteal layers at any rate must be preserved. It should also be remembered that Ollier had very little practice in the manifestations of the totally diaphyseal resections by the sub-periosteal method.

When the osteitis is already fairly pronounced, the speaker said that the raised periosteum presented the appearance, to the eye and to the finger, of the tongue of a cat; that is to say, the surgeon has left all the heads of the little growths productive of ostitis, which comes from a cortical growth and not from a sub-periosteal formation.

It is no longer a case of using the ossifying power of an osteogenous layer belonging to the periosteum and irritated by inflammation; the question is rather to leave to the inner surface of the periosteum sufficient bone tissue to generate new bone substance. But for quite a long time surgeons have found out that, in practice, when the periosteum is detached with a rugine, the superficial ossifiable elements remain, on the whole, adherent to the periosteum.

The real osteogenous layer would be the upper surface of the bone itself.

